# The efficacy of hypotonic and near-isotonic saline for parenteral fluid therapy given at low maintenance rate in preventing significant change in plasma sodium in post-operative pediatric patients: protocol for a prospective randomized non-blinded study

**DOI:** 10.1186/1471-2431-11-61

**Published:** 2011-07-05

**Authors:** Urban Fläring, Per-Arne Lönnqvist, Björn Frenckner, Jan F Svensson, Ingimar Ingolfsson, Lena Wallensteen, Shayarina Stigzelius, Jan Kowalski, Rafael T Krmar

**Affiliations:** 1 Department of Pediatric Anesthesia and Intensive Care, Astrid Lindgren Children's Hospital, Karolinska University Hospital, Solna, S-171 76 Stockholm, Sweden; 2 Department of Pediatric Surgery, Astrid Lindgren Children's Hospital, Karolinska University Hospital, Solna, S-171 76 Stockholm, Sweden; 3 Karolinska Institutet, Department for Clinical Science, Intervention and Technology, Division of Pediatrics, Karolinska University Hospital, Huddinge, S-141 86 Stockholm, Sweden

## Abstract

**Background:**

Hyponatremia is the most frequent electrolyte abnormality observed in post-operative pediatric patients receiving intravenous maintenance fluid therapy. If plasma sodium concentration (p-Na^+^) declines to levels below 125 mmol/L in < 48 h, transient or permanent brain damage may occur. There is an intense debate as to whether the administered volume (full rate *vs. *restricted rate of infusion) and the composition of solutions used for parenteral maintenance fluid therapy (hypotonic *vs. *isotonic solutions) contribute to the development of hyponatremia. So far, there is no definitive pediatric data to support a particular choice of parenteral fluid for maintenance therapy in post-surgical patients.

**Methods/Design:**

Our prospective randomized non-blinded study will be conducted in healthy children and adolescents aged 1 to 14 years who have been operated for acute appendicitis. Patients will be randomized either to intravenous hypotonic (0.23% or 0.40% sodium chloride in glucose, respectively) or near-isotonic (0.81% sodium chloride in glucose) solution given at approximately three-fourths of the average maintenance rate. The main outcome of interest from this study is to evaluate 24 h post-operatively whether differences in p-Na^+ ^between treatment groups are large enough to be of clinical relevance. In addition, water and electrolyte balance as well as regulatory hormones will be measured.

**Discussion:**

This study will provide valuable information on the efficacy of hypotonic and near-isotonic fluid therapy in preventing a significant decrease in p-Na^+^. Finally, by means of careful electrolyte and water balance and by measuring regulatory hormones our results will also contribute to a better understanding of the physiopathology of post-operative changes in p-Na^+ ^in a population at risk for hyponatremia.

**Trial registration:**

The protocol for this study is registered with the current controlled trials registry; registry number: ISRCTN43896775.

## Background

Hyponatremia, as defined by plasma sodium concentration (p-Na^+^) < 136 mmol/L, is the most common electrolyte abnormality in post-operative pediatric patients receiving parenteral maintenance fluid therapy [1]. Patients with hospital-acquired hyponatremia are exposed to major neurological complications if p-Na^+ ^declines to ~ 120 mmol/L in < 48 h [2].

Post-operative hyponatremia has been attributed to either an excess of delivered water or a net sodium deficit [1,3-5]. A series of pediatric investigations support the notion that a hospital acquired decrease in p-Na^+ ^in ill children receiving parenteral fluid therapy is the result of administering an excess of water relative to sodium, i.e., hypotonic saline solutions [6,7]. However, there is no definitive pediatric data to support a particular choice of parenteral fluid for maintenance therapy to prevent hyponatremia in either post-surgical patients or critically ill medical patients [8-10]. Therefore, data obtained by means of well-designed randomized clinical trials remains a pressing priority if uncertainties in the prescription of parenteral maintenance fluid therapy are to be resolved [11,12]. Based on previous lines of evidence, such an investigation should take into account the following facts: *i) *healthy kidneys are able to greatly vary the amount of water and sodium excreted to match the water and salt intake and therefore preserve the stability of the extracellular fluid compartment and maintain p-Na^+ ^within the physiological range [13,14], *ii) *conditions such as stress (e.g., pain, fever, and surgery), hypovolemia, vomiting, nausea, anesthesia, and intra-operative bowel manipulation are known non-osmotic stimuli for the production of antidiuretic hormone (ADH) [3,15-17], and *iii) *increased ADH levels impair the urinary dilution capacity and as a result free water retention in excess of sodium may ensue [18,19].

Taking this information into account, prescription of parenteral fluid therapy following a surgical intervention might pose a risk for a decrease in p-Na^+ ^whenever the kidneys lack the capacity to excrete an excess of water load [20,21]. Under such circumstances, hyponatremia would be the result of dilution of body solutes, i.e., an excess of water in relation to the existing sodium stores rather than to an increase in urinary sodium excretion. This suggests that after surgery, the prescription of parenteral maintenance fluid therapy should be tailored to the patient's water and electrolyte needs [22]. Indeed, in recent years it has been recommended to prescribe half the average of daily physiological losses as a measure to prevent post-operative hyponatremia [12,23,24]. This recommendation appears to be appropriate whenever the patient's extracellular fluid compartment is preserved and the urinary dilution capacity is suspected to be impaired by non-osmotic stimulated ADH activity [8].

Conversely, there is data supporting the view that hyponatremia might ensue due to desalination followed by the administration of intravenous replacement with hypotonic fluids [23]. It has been proposed that post-operative hyponatremia would be avoided by using isotonic saline instead of hypotonic saline for parenteral maintenance fluid therapy [11,25]. There is however no consensus as to whether or not the isotonic saline infusion rate should be restricted [11,25]. Notwithstanding this, a fall in p-Na^+ ^has also been also observed in surgical patients receiving near-isotonic saline infusions [26].

Based on existing pediatric data, it appears that tailoring post-operative parenteral fluid therapy to the individual patient's needs would be the most appropriate physiological approach, since no simple parenteral fluid therapy seems likely to be safely used in this clinical setting. Nevertheless, there is so far no study that suggests how this should be done.

At our hospital, the routine procedure for immediate post-operative parenteral maintenance fluid therapy consists of the use of near half-isotonic solution (0.40% salt solution, 70 mmol/L Na^+ ^+ 45 mmol/L Cl^- ^+ 25 mmol/L acetate in 2.5% glucose) given at low maintenance rate, i.e., at ~¾ the average maintenance rate. The administration of sodium acetate is intended as an alternative to NaCl as a source of sodium ion in a large volume infusion. In addition, acetate is an alternative source of bicarbonate by metabolic conversion in the liver. Although in our own experience (UF and PAL, unpublished data), no patient treated in this way has experienced hyponatremia of potential clinical significance, defined as a fall in p-Na^+ ^fall < 130 mmol/L [27], our routine procedure does not rely on evidence-based medicine, but on theoretical grounds. Therefore, a well designed study seems warranted to determine the parenteral fluid regimen that can effectively and accurately be prescribed to post-operative pediatric patients [28]. In addition, logic-based evidence for appropriate laboratory monitoring is also justified.

In this paper, we detail our study protocol for a prospective randomized non-blinded investigation aimed at evaluating the efficacy of hypotonic and near-isotonic saline for parenteral fluid therapy given at low maintenance rate in post-operative pediatric patients, using p-Na^+ ^as a surrogate marker. Since at our Center acute appendicitis accounts for a large number of emergency surgical admissions, our investigation will be conducted in previously healthy children and adolescents who have undergone acute appendectomy for complicated appendicitis. The study will be started once the rationale behind this investigation as well as the full description of the protocol has reached the public domain. In so doing, we expect to receive comments that would eventually contribute to improve our protocol [29].

## Methods

### Objectives

Our primary outcome of interest is to evaluate whether the post-operative prescription of either hypotonic or near-isotonic fluid therapy given at low maintenance rate in otherwise healthy children with normal post-operative p-Na^+ ^are equally effective in maintaining p-Na^+ ^within the normal range. In addition, our secondary aim is to determine water and electrolyte-balance following the administration of the investigational parenteral maintenance fluids as well as to investigate known regulatory hormones involved in the homeostatic control of water balance. Finally, we will seek to determine how frequently p-Na^+ ^should be monitored during the post-operative period.

### Study Design

The present investigations will be set up as a prospective randomized non-blinded study. Eligible patients will be randomly assigned to receive one of the following electrolyte/glucose combinations as parenteral maintenance fluid therapies during the 24 h following surgery: *i) *0.23% sodium chloride (40 mmol/L Na^+ ^+ 20 mmol/L K^+ ^+ 60 mmol/L Cl^-^; extempore solution) in 5% glucose, *ii) *0.40% sodium chloride (70 mmol/L Na^+ ^+ 45 mmol/L Cl^- ^+ 25 mmol/L acetate; B. Braun Melsungen AG, Melsungen, Germany) in 2.5% glucose, or *iii) *0.81% sodium chloride (140 mmol/L Na^+ ^+ 4 mmol/L K^+ ^+ 1 mmol/L Ca^2+ ^+ 1 mmol/L Mg^2+ ^+ 118 mmol/L Cl^- ^+ 30 mmol/L acetate; Serumwerk Bernburg AG, Bernburg, Germany) in 1% glucose (the chemical symbols K^+^, Cl^-^, Ca^2+^, and Mg^2+ ^refer to potassium, chloride, calcium, and magnesium, respectively).

### Ethical Considerations

The study protocol has been approved by the Medical Ethics Committee at Karolinska University Hospital, Stockholm, Sweden (Protocol No. 2010/2:9). The study protocol was registered at current clinical trials registry database; http://www.controlled-trials.com/ISRCTN43896775, registry number: ISRCTN43896775).

A written and verbal version of the information will be presented to the potentially eligible patient and parents before the surgical procedure by the surgeon in charge of the patient. The exact nature of the study, the implications and constraints of the protocol, the known side effects, and any risks involved in taking part will be detailed. Parents and patients will be told that all study findings will be handled in strictest confidentiality and that it extends beyond the duration of the study. It will be clearly stated that the patient and parents are free to withdraw from the study at any time for any reason without prejudice to future care, and with no obligation to give the reason for withdrawal. Written informed consent will then be obtained by means of a dated patient's and parent's signature and dated signature of the surgeon who presented the informed consent form. A copy of the signed informed consent will be given to the patient.

### Study Population and Site

The participants will be recruited at the Department of Pediatric Surgery, Astrid Lindgren Children's Hospital, at the Karolinska University Hospital, Solna, Sweden. Potentially eligible participants in the study will be healthy children and adolescents consecutively operated for emergency abdominal surgery due to acute appendicitis.

Our Center is an academic teaching hospital that provides care in the majority of pediatric surgical cases in the greater Stockholm area, covering a population of about 1,8 million inhabitants, which corresponds to a referral area of approximately 350,000 children. More than 300 children and adolescents are admitted for treatment of acute appendicitis every year. The date for enrolment to the clinical trial will be defined once the protocol has reached public domain. The study will take place in the Pediatric Intensive Care Unit, Astrid Lindgren Children's Hospital, at the Karolinska University Hospital, Solna, Sweden.

### Eligibility Criteria

In the present investigation, we arbitrarily decided not to include children aged less than 1 year of age because the functional capacity of the kidney, including glomerular filtration rate and renal concentration ability, as well as the efficiency to excrete sodium load is lower in infants than in older children [30-33]. Hence, the study population will be male and female children and adolescents aged between 1 and 14 years old whose post-operative p-Na^+ ^are within the normal range (local reference range: 135 - 147 mmol/L). Children and adolescents undergoing emergency intervention for treatment of acute appendicitis either as an open or laparoscopic procedure will be considered to be potentially eligible. To qualify for randomization, the participants are required to have a complicated appendicitis, i.e., gangrenous or perforated, either with generalized or localized peritonitis of such a degree that the surgeon considers oral feeding unsuitable for at least 24 h following the surgery. Consequently, the randomization will be performed post-operatively and on the basis of intra-operative findings.

We infer that in our study population, the prescription of parenteral fluid therapy for the immediate 24 h post-operative period might be justified, and that by delaying oral fluid therapy intake, in any particular case, will do no harm to the patient.

The exclusion criteria will be any clinical condition, such as renal disease, acute or chronic lung inflammation, pituitary or hypothalamic disease, and adrenal insufficiency that eventually can lead to electrolyte and water imbalance. In addition, any patient whose p-Na^+ ^is < 130 mmol/L after the initiation of parenteral maintenance fluid therapy, will be withdrawn from the study and treated accordingly at the discretion of the anesthesiologist responsible for the patient's care [34].

Whenever the occurrence of vomiting is reported or if the patient complains of symptoms attributable to symptomatic hyonatremia such as nausea, malaise, headache, restlessness, drowsiness, or decreased consciousness, p-Na^+ ^will be immediately controlled and the event clearly documented in the medical record.

During the first 24 h post-operatively, the administration of extra fluid besides the study maintenance fluid therapy, will be not permitted with the exception of fluid administered with medication or to maintain patency of peripheral intravenous catheters.

### Analysis population

Three analysis populations will be used in this study. The "intention to treat" population will consist of all subjects who have been randomized and have received one of three parenteral maintenance fluid therapies previously mentioned. The analysis will be conducted in all "intention to treat" patients who had no protocol violation(s) or deviation(s).

### Patient Randomization

The study population will be allocated to each of the intervention groups using randomization in blocks. Block size will be kept secret to all study related personnel. Participants will be numbered sequentially. It is expected that after the randomization there will be no differences in the clinical or biochemical characteristics among different treatment groups at the start of post-operative parenteral fluid therapy. After randomization, the anesthetist and the clinical team will be informed by the surgeon to which fluid regimen arm the patient has been allocated.

### Clinical and Laboratory Assessment

Upon admission from surgery and by the end of the first post-operative day, patients will be weighted (kg). In addition, height (m) will be measured and body mass index (kg/m^2^) calculated. Non-invasive blood pressure (mm Hg), heart rate (beats per minute), monitoring of arterial oxygen saturation with pulse oximetry, and body temperature will be recorded at regular intervals.

Blood samples will be collected immediately after the conclusion of surgery (T_0_), at 6 h (T_6_) and 12 h (T_12_), and then at 24 h (T_24_) following the surgery. The blood samples obtained at T_0 _and T_24 _will be analyzed for plasma (p) Na^+^, K^+^, Cl^-^, creatinine, urea, uric acid, glucose, lactate, ketones, renin, aldosterone, ADH, hemoglobin, blood gas, and for serum (s) osmolality by means of routine and quality controlled laboratory techniques. Serum Cortisol and s-TSH (thyroid-stimulating hormone) will be measured at T_0_. At T_12_, p-Na^+^, p-glucose, and blood gas will be monitored. The blood samples obtained at T_6 _and T_12 _will be analyzed for p-Na^**+**^. Also, at the discretion of the treating anesthesiologist, p-Na^+ ^will be monitored at intermediate sampling if p-Na^+ ^at T_12 _follows a decreasing trend.

Entire urine collection from T_0 _to T_24 _will be obtained by urine bag or clean catch, as appropriate. A bladder scan will be performed whenever urinary retention is suspected. Depending on the bladder volume measured, urethra catheterization will be performed to relieve urine retention if the patient cannot void on his/her own. Adequacy of timed urine collection will be confirmed as far as possible by estimation of urinary creatinine excretion rate (mmol/kg per 24 h) [35].

In addition to measuring total urine volume, urinary sodium, potassium, and chloride excretion, we will calculate, from spot urine samples obtained at T_0 _and T_24_, the fractional excretion of sodium (FeNa), the fractional excretion of uric acid (FeUA), and the urine osmolality.

The FeNa [(u-Na^+ ^× p-creatinine) ÷ (p-Na^+ ^× u-creatinine) × 100] is the fraction of sodium filtered by the glomeruli that appears in the urine. The extracellular fluid compartment is regulated by changes in sodium excretion and when the extracellular fluid is contracted (e.g., intra-operative hypovolemia due to blood loss), urinary sodium can be undetectable. The FeNa is therefore a useful laboratory investigation to differentiate hypovolemic, i.e., a decrease of the effective arterial blood volume, from euvolemic state [36]. On the other hand, hypervolemic state enhances FeNa, an effect independent of the increase in urinary flow.

In the event of non-osmotic excess secretion of ADH, as it has been observed in post-operative patients, generous intake of solute-poor fluid might result in a positive water balance. If post-operative fluid prescription is however limited, as we intend in our study, the extracellular fluid compartment (and total body fluid) will not be expected to expand. Consequently, the FeNa at T_24 _should not be significantly different compared to T_0_.

Uric acid urinary excretion is regulated to changes in the effective arterial blood volume [37]. Therefore, FeUA [(u-UA × p-creatinine) ÷ (p-UA × u-creatinine) × 100] is also useful to indirectly infer the effective arterial blood volume, provided that the renal proximal tubule is properly functioning. This and the aforementioned laboratory investigations will provide an insight into how a child's kidneys are handling water and salt.

Urine analysis, including osmolality, will be performed by using routine and quality controlled techniques. Taken together, FeNa, FeUA, total urinary electrolyte excretion, urine osmolality, ADH, as well as p-renin and p-aldosterone activity will be interpreted in the context of the patient's water and electrolyte balance.

Finally, serum (~3 ml), and urine samples (~15 ml), obtained at T_0 _and T_24 _will be stored -80°C in a biobank (duration of storage 10 years) for eventual future analysis.

### Parenteral Fluid Therapy

*Intra-operative fluid management*: following our routine procedure, the volume of intra-operative fluid will be adapted to the severity of surgical trauma as follows; *i) *mild trauma, 5 ml/kg body weight (BW) per h, *ii) *moderate trauma, 7.5 ml/kg BW per h, and *iii) *severe trauma, 10 ml/kg BW per h. The parenteral fluid used intra-operatively, i.e., from the start of the surgery to T_0_, will consist of 0.40% salt solution (70 mmol/L Na^+ ^+ 45 mmol/L Cl^- ^+ 25 mmol/L acetate) in 2.5% glucose (B. Braun Melsungen AG, Melsungen, Germany).

During surgery, appropriate amount of intravenous isotonic fluid blouses will be administered whenever hypovolemia is suspected to ensure adequate intravascular volume and oxygen delivery. The duration of anesthesia and all intra-operative inputs will be documented in the medical record. In addition, we will have free and open access to data concerning patient's pre-operative intravenous fluid therapy.

*Post-operative fluid management*: the fluid needs for parenteral therapy will be estimated based on metabolic requirements of the body, i.e., expenditure of energy, as described by Holliday and Segar where daily caloric expenditure is equal to 100 cal/kg BW up to 10 kg, 1000 cal + 50 cal/kg BW from 11 to 20 kg, and 1500 cal + 20 cal/kg BW over 20 kg [22]. Under normal conditions, the average needs of water for replacement of usual insensible and urinary water losses is directly related to the metabolic rate, and when expressed in milliliters, equals the estimated daily energy expenditure in calories [22]. Depending on age, the amount of water that accounts for insensible water losses varies from 45 to 59 ml/100 cal per 24 h, while the replacement of urinary water losses will depend on the renal solute load, i.e., the total daily excretion of solutes, and the concentration of these solutes in the urine [22]. Holliday and Segar estimated that a solute load of 40 mosmol/100 cal per 24 h, a figure expected in the instance of rational parenteral fluid therapy, would require ~60 ml of water to be excreted, i.e., 60 ml/100 cal per 24 h. This figure was calculated at a urine osmolality of 600 mosmol/kg H_2_0, which was considered the maximum to safely be expected from patients receiving parenteral fluid therapy under normal conditions [22].

Since prescription of intravenous fluid therapy given at full maintenance rate may be potentially dangerous when renal water excretion is limited by ADH excess, parenteral fluid therapy will be given at a lower rate than the recommended average maintenance rate [8]. Consequently, and, in accordance with our local care guidelines for parenteral fluid therapy following surgical procedures, ~¾ of the average maintenance rate will be prescribed for the first 24 h post-operatively, i.e., from T_0 _to T_24_. Table 1 relates body weight to ~¾ of the average maintenance allowances for hourly periods.

**Table 1:** 24 h post-operative fluid volume at ~¾ of average maintenance rate according to Holliday-Segar formula.

Weight (kg)	ml/kg/24 h
10	80
11	76
12	73
13	71
14	69
15	67
16	65
17	64
18	62
19	61
20	60
22	56
24	53
26	51
28	49
30	47
35	43
40	40
45	38
50	36
55	35
60	33
65	32
70	31

Ongoing post-operative losses, e.g., nasogastric drainage following abdominal surgery, will be replaced ml for ml with Ringer's lactate solution.

Below, we describe an example of the likely change in p-Na^+ ^that would result at T_24 _in a previously healthy 10 year-old-boy who met the inclusion criteria to participate in the study. We presuppose that our hypothetical case has been randomized to be treated with 0.23% salt solution in 5% glucose. We assume that, similar to patients we expect will participate in our study, his kidneys are capable of generating concentrated urine in response to non-osmotic stimulated ADH activity. Since in the immediate post-operative period a child with intact urinary concentration ability may achieve urine osmolality of more than 700 mosmol/kg H_2_O as a result of elevated ADH levels [38], in our example we arbitrarily set the urine osmolality at 900 mosmol/kg H_2_O.

In this hypothetical case we take into account all the patient's inputs during the 24 h period following surgery, i.e., total volume of water, Na^+^, K^+^, and Cl^-^. Each element is analyzed separately and it is supposed that all the infused Na^+^, K^+^, and Cl^- ^represent the renal solute load that eventually will be excreted in the urine [39]. Thus, one mmol of each of these elements equals one mosmol. Since glucose is metabolized to carbon dioxide and water, it does not contribute to renal solute load in our example. Similarly, in solutions containing acetate, this anion is metabolized in the liver and does not contribute to renal solute load.

It should be noted that, for the sake of simplicity, we have chosen to take for granted that in this hypothetical case the urine osmolality would be fixed during the 24 h period following surgery. This, and the fact we only consider the patient's input of water and electrolyte, might confine the accuracy in our predicting p-Na^+ ^at T_24_. Therefore, in our study, the patient's expected p-Na^+ ^at T_24 _will be calculated by computing actual water and electrolyte balance during the first 24 h post-operatively as follows:

where the subscripts _f _and _i _represents final (T_24_) and initial (T_0_) p-Na^+^, respectively; Δ (Na^+ ^+ K^+^) represents cation input (infused) minus cation output (urinary excretion); TBW represents the estimated initial total body water and ΔTBW describes the water infused minus water excreted, respectively.

In our study we will analyze, by applying the same approach as described in our hypothetical case, whether p-Na^+ ^at T_24 _can be correctly estimated by using the patient's immediate postoperative urine osmolality value. The estimated p-Na^+ ^obtained by this method and by means of water and electrolyte balance will be plotted against the patient's actual p-Na^+ ^measured at T_24 _for assessing agreement between methods [40].

Twenty-four hour insensible water losses in each participant will be calculated as follows:

### Example

Weight = 30 kg. P-Na^+ ^at T_0 _= 140 mmol/L.

24 h infused volume according to study protocol (Table 1) = 1410 ml.

24 h insensible (~45 ml/100 cal per 24 h) = 765 ml [22].

The expected 24 h urine output needed to maintain water balance will be as follows: infused volume - insensible losses = 645 ml.

By infusing 0.23% salt solution (40 mmol/L Na^+ ^+ 20 mmol/L K^+ ^+ 60 mmol/L Cl^-^) in 5% glucose, the electrolyte intake (and therefore excretion) will be expected to be as follows:

At a fixed urine osmolality of 900 mosmol/kg H_2_O, because of hypothetically persistent ADH release, our example case will be expected to produce 188 ml urine per 24 h (i.e., solute load of 169.2 mosmol ÷ urine osmolality of 900 mosmol/kg H_2_O) [22]. As a consequence, 457 ml of water will be retained (645 ml - 188 ml); ⅔ of this will distribute to the intracellular fluid compartment and ⅓ to the extracellular fluid compartment [41]. Since the apparent volume of distribution of sodium is the total body water (TBW) [42], and the estimated TBW accounts for 60% of body weight [43], the expected change in p-Na^+ ^can be treated algebraically as follows:

where the subscripts _i _and _f _represent initial and final states, respectively. This equation states that the initial volume × initial concentration = final volume × final concentration. Since we assume that in our hypothetical case there will be no change in total body sodium content by the end of the first postoperative 24 h, the p-Na^+^_f _is now the original sodium content divided by TWB_f_. Thus, in our example the p-Na^+^_f _will be expected to be 136.5 mmol/L (i.e., p-Na^+^_f _= 18 L × 140 mmol/L ÷ 18.457 L).

If we replace 0.23% sodium chloride parenteral fluid therapy with either 0.40% sodium chloride or 0.81% sodium chloride, the expected p-Na^+^_f _would be 136.4 mmol/L and 138.2 mmol/L, respectively.

#### Sample size calculation

The expected sample size required in our study is based on the calculation for equality applied to the primary outcome variable p-Na^+^, measured at T_24 _as mmol/L.

In the previous section we estimated that the largest expected fall in p-Na^+ ^in our illustrative case would be 3.6 mmol/L. Consequently, if we apply this figure to a patient whose p-Na^+ ^at T_0 _is 135 mmol/L, the expected p-Na^+ ^at T_24 _after giving 0.4% sodium chloride would be 131.4 mmol/L, a plasma level which may not have clinical significance [27]. However, we are compelled to speculate that this figure may not be exact because in our illustrative case we dealt with hypothetical data that may not necessary mirror the clinical setting. Hence, our estimate has to be verified by means of water and electrolyte balance measurement *in vivo*. Taken this uncertainty into account, we arbitrarily decided that the equality margin will be set at 5 mmol/L. This value will capture our estimate, but, most importantly, in patients whose p-Na^+ ^at T_0 _is 135 mmol/L, it would allow us to identify those patients who are at risk for developing hyponatremia of potential clinical significance [27]. Thus, a difference between treatment groups greater than this margin will be considered as clinically relevant. Consequently, a difference smaller than 5 mmol/L will be considered as clinically less relevant. Sample size was calculated assuming a standard deviation (SD) of 5 mmol/L, a significance level of 0.05, and a power of 80% to detect a difference larger than the equality margin of 5 mmol/L. Hence, the number of patients per study arm needed to fulfill the power goal of the study will be 17 patients.

#### Data analysis

All statistical analyses will be described in detail in the statistical analysis plan. Data will be presented using descriptive statistics, i.e., mean and SD for continuous variables, and frequency and proportions for categorical variables. The efficacy variable will be evaluated using ANCOVA, intervention (parenteral maintenance fluid therapy) as a fixed factor and baseline levels as a covariate in the model. The corresponding 95% confidence intervals for each group and the difference between groups will also be presented. In addition, graphs will be used in order to facilitate the interpretation of the results regarding the equality margins to be used in the present study.

Missing data will be replaced using a conservative approach, which will be specified in the statistical analysis plan. All tests will be two-sided and performed at the significance level of 0.05.

## Discussion

Post-operative symptomatic hyponatremia in patients receiving intravenous maintenance fluid therapy may be associated with severe cerebral dysfunction and high mortality [44]. Our study intends therefore to address a question that might have important clinical implications. To put our study question into perspective, we summarize the results of the most relevant pediatric studies published so far on this topic.

A retrospective single-center study reported on the incidence and clinical consequences of post-operative hyponatremia in children [45]. Out of 145 pediatric patients who met the criteria for further analysis, 16 children were identified as having had p-Na^+ ^< 130 mmol/L during the 24 h following the surgical procedure. In the post-operative period, the incidence of hyponatremia was similar between patients receiving hypotonic and isotonic fluid therapy. None of them experienced neurological sequelae due to hyponatremia. According to the data presented in this study, and by excluding one patient because of pseudohyponatremia, it seems that in 14 of the 15 patients the rate of infusion of parenteral fluid therapy given over the 24 h following the surgical procedure was almost equal (*n *= 8), equivalent (*n *= 4), or higher (*n *= 2) than the average maintenance fluid therapy that would otherwise be recommended to healthy children under normal clinical conditions [22]. This observation suggests that the volume and not the composition of intravenous solutions used for parenteral fluid therapy might have accounted for the development of post-operative hyponatremia.

One study, including medical and surgical patients followed from 12 to 24 h after admission, found that the prescription of 0.18% sodium chloride in 5% glucose (30 mmol/L Na^+^) was associated with a significant fall in p-Na^+ ^when compared with patients who received isotonic saline (0.9% sodium chloride, 154 mmol/L Na^+^) [6]. The incidence of hyponatremia was not reported. Full maintenance parenteral fluid therapy was found to produce a greater but not significant fall in p-Na^+ ^than the fluid therapy administered at a restricted rate.

A separate investigation, also designed as a randomized controlled study, conducted in critically ill medical and surgical patients, showed that at 24 h of starting full maintenance parenteral fluid therapy the fall in p-Na^+^, often to mild hyponatremic levels, was higher in patients receiving fluids with a sodium content < 100 mmol/L compared with patients receiving intravenous solutions containing 140 mmol/L Na^+ ^[7].

Although the aforementioned investigations were designed to provide the most reliable form of scientific evidence, they have some limitations that weaken the power of the studies, e.g., study population heterogeneity, wide age-range groups, no water and electrolyte balance, no measurement of regulatory hormones, no standardization of fluid therapy given intra-operatively, and short follow-up. Still, these investigations are important since they indicate that a hospital-acquired decrease of p-Na^+ ^in patients receiving parenteral maintenance fluid therapy may be avoidable.

A recent randomized trial comparing multiple post-operative intravenous maintenance fluid treatment groups determined the risk of hyponatremia in patients aged between 6 months and 15 years admitted for either elective or emergency surgery [46]. The authors observed that 24 h after surgery, regardless of fluid rate, i.e., either 100% or 50% maintenance fluid rate, there was no difference in the incidence of hyponatremia between patients who received 0.9% sodium chloride and 0.45% sodium chloride. On multiple linear regression analysis, hypotonic fluid, but not rate, significantly influenced the change in p-Na^+ ^from induction of anesthesia to 24 h after surgery. It is important to observe, that at this time-point after surgery, no patient in either treatment arm group experienced a fall in p-Na^+ ^below 130 mmol/L. This study did not report on both the total intravenous volume prescribed after surgery and urinary production, making therefore difficult to analyze the role of water and electrolyte balance on p-Na^+ ^values at 24 h after surgery.

Finally, in a previous prospective study investigating the incidence of post-operative hyponatremia in pediatric patients who uniformly received hypotonic parenteral fluid therapy at full maintenance rate [47], the prevalence of hyponatremia, defined as p-Na^+ ^< 136 mmol/L, was 21% and 31% at 12 h and at 24 h following surgery, respectively. Of note, no patient experienced a decrease in p-Na^+ ^below 130 mmol/L, suggesting that the inclusion of an hypotonic saline administration arm in future clinical trials is a feasible option [48].

In the present paper we describe the objectives of our study and the design to achieve them. Since no consensus has been reached on post-operative fluid management, the results of this investigation will contribute to the evidence base for effective pediatric prescription of parenteral maintenance fluid therapy in otherwise healthy children and adolescents undergoing acute surgery. Although our investigation will be conducted in a homogeneous cohort of patients, we speculate that our results would be of clinical importance, not only in post- appendectomized children and adolescents but also in other post-operative scenarios.

## Competing interests

The authors declare that they have no competing interests.

## Authors' contributions

UF, PAL, BF, and RTK are responsible for general study design. JFS, II, LW and SS will assemble input data and co-supervise the study. JK planned the statistical analysis. RTK conceived the study, wrote the manuscript and will act as guarantor for the paper. All the authors critically reviewed the manuscript for scientific content and approved the final version.

## Pre-publication history

The pre-publication history for this paper can be accessed here:

http://www.biomedcentral.com/1471-2431/11/61/prepub

## References

[B1] Halberthal M Halperin ML Bohn D Lesson of the week: Acute hyponatraemia in children admitted to hospital: retrospective analysis of factors contributing to its development and resolution BMJ 2001 322 7289 780 782 10.1136/bmj.322.7289.780 11282868 PMC1119957

[B2] Arieff AI Hyponatremia, convulsions, respiratory arrest, and permanent brain damage after elective surgery in healthy women The New England journal of medicine 1986 314 24 1529 1535 10.1056/NEJM198606123142401 3713746

[B3] Adrogue HJ Madias NE Hyponatremia The New England journal of medicine 2000 342 21 1581 1589 10.1056/NEJM200005253422107 10824078

[B4] Durward A Tibby SM Murdoch IA Hyponatraemia can be caused by standard fluid regimens BMJ 2000 320 7239 943 10.1136/bmj.320.7239.943 10742022 PMC1117857

[B5] Bohn D Children are another group at risk of hyponatraemia perioperatively BMJ 1999 319 7219 1269 10550107 10.1136/bmj.319.7219.1269 PMC1117036

[B6] Yung M Keeley S Randomised controlled trial of intravenous maintenance fluids J Paediatr Child Health 2009 45 1-2 9 14 10.1111/j.1440-1754.2007.01254.x 18036144

[B7] Montanana PA Modesto i Alapont V Ocon AP Lopez PO Lopez Prats JL Toledo Parreno JD The use of isotonic fluid as maintenance therapy prevents iatrogenic hyponatremia in pediatrics: a randomized, controlled open study Pediatr Crit Care Med 2008 9 6 589 597 10.1097/PCC.0b013e31818d3192 18838929

[B8] Holliday MA Ray PE Friedman AL Fluid therapy for children: facts, fashions and questions Arch Dis Child 2007 92 6 546 550 10.1136/adc.2006.106377 17175577 PMC2066164

[B9] Choong K Kho ME Menon K Bohn D Hypotonic versus isotonic saline in hospitalised children: a systematic review Arch Dis Child 2006 91 10 828 835 10.1136/adc.2005.088690 16754657 PMC2066024

[B10] Bailey AG McNaull PP Jooste E Tuchman JB Perioperative crystalloid and colloid fluid management in children: where are we and how did we get here? Anesth Analg 110 2 375 390 10.1213/ANE.0b013e3181b6b3b5 19955503

[B11] Moritz ML Ayus JC Prevention of hospital-acquired hyponatremia: a case for using isotonic saline Pediatrics 2003 111 2 227 230 10.1542/peds.111.2.227 12563043

[B12] Holliday MA Segar WE Friedman A Reducing errors in fluid therapy management Pediatrics 2003 111 2 424 425 12563072

[B13] Carlotti AP Bohn D Mallie JP Halperin ML Tonicity balance, and not electrolyte-free water calculations, more accurately guides therapy for acute changes in natremia Intensive Care Med 2001 27 5 921 924 10.1007/s001340100911 11430551

[B14] Coulthard MG Will changing maintenance intravenous fluid from 0.18% to 0.45% saline do more harm than good? Arch Dis Child 2008 93 4 335 340 10.1136/adc.2007.124461 18356386

[B15] Halperin ML Bohn D Clinical approach to disorders of salt and water balance. Emphasis on integrative physiology Crit Care Clin 2002 18 2 249 272 10.1016/S0749-0704(01)00008-2 12053833

[B16] Moritz ML Ayus JC Disorders of water metabolism in children: hyponatremia and hypernatremia Pediatr Rev 2002 23 11 371 380 12415016

[B17] Guy AJ Michaels JA Flear CT Changes in the plasma sodium concentration after minor, moderate and major surgery Br J Surg 1987 74 11 1027 1030 10.1002/bjs.1800741123 3690229

[B18] Segar WE Moore WW The regulation of antidiuretic hormone release in man: I. Effects of change in position and ambient temperature on blood ADH levels J Clin Invest 1968 47 9 2143 2151 10.1172/JCI105900 16695953 PMC297375

[B19] Judd BA Haycock GB Dalton N Chantler C Hyponatraemia in premature babies and following surgery in older children Acta Paediatr Scand 1987 76 3 385 393 10.1111/j.1651-2227.1987.tb10487.x 3604658

[B20] Leaf A Bartter FC Santos RF Wrong O Evidence in man that urinary electrolyte loss induced by pitressin is a function of water retention J Clin Invest 1953 32 9 868 878 10.1172/JCI102805 13084753 PMC438416

[B21] Verbalis JG Pathogenesis of hyponatremia in an experimental model of the syndrome of inappropriate antidiuresis Am J Physiol 1994 267 6 Pt 2 R1617 1625 7810773 10.1152/ajpregu.1994.267.6.R1617

[B22] Holliday MA Segar WE The maintenance need for water in parenteral fluid therapy Pediatrics 1957 19 5 823 832 13431307

[B23] Arieff AI Ayus JC Fraser CL Hyponatraemia and death or permanent brain damage in healthy children BMJ 1992 304 6836 1218 1222 10.1136/bmj.304.6836.1218 1515791 PMC1881802

[B24] Holliday MA Friedman AL Segar WE Chesney R Finberg L Acute hospital-induced hyponatremia in children: a physiologic approach The Journal of pediatrics 2004 145 5 584 587 10.1016/j.jpeds.2004.06.077 15520753

[B25] Taylor D Durward A Pouring salt on troubled waters Arch Dis Child 2004 89 5 411 414 10.1136/adc.2003.045302 15102626 PMC1719903

[B26] Steele A Gowrishankar M Abrahamson S Mazer CD Feldman RD Halperin ML Postoperative hyponatremia despite near-isotonic saline infusion: a phenomenon of desalination Ann Intern Med 1997 126 1 20 25 8992919 10.7326/0003-4819-126-1-199701010-00003

[B27] Fraser CL Arieff AI Epidemiology, pathophysiology, and management of hyponatremic encephalopathy The American journal of medicine 1997 102 1 67 77 10.1016/S0002-9343(96)00274-4 9209203

[B28] Lonnqvist PA Inappropriate perioperative fluid management in children: time for an isotonic solution?! Author's reply Paediatr Anaesth 2008 18 3 271 18230074 10.1111/j.1460-9592.2007.02356.x

[B29] Altman DG The scandal of poor medical research BMJ 1994 308 6924 283 284 8124111 10.1136/bmj.308.6924.283 PMC2539276

[B30] Rubin MI Bruck E Rapoport M Snively M McKay H Baumler A Maturation of Renal Function in Childhood: Clearance Studies J Clin Invest 1949 28 5 Pt 2 1144 1162 10.1172/JCI102149 16695787 PMC439672

[B31] Aperia A Broberger O Thodenius K Zetterstrom R Development of renal control of salt and fluid homeostasis during the first year of life Acta Paediatr Scand 1975 64 3 393 398 10.1111/j.1651-2227.1975.tb03853.x 1155057

[B32] Kleinman LI Developmental renal physiology Physiologist 1982 25 2 104 110 7089080

[B33] Marild S Jodal U Jonasson G Mangelus L Oden A Persson NG Reference values for renal concentrating capacity in children by the desmopressin test Pediatric nephrology (Berlin, Germany) 1992 6 3 254 257 10.1007/BF00878361 1616834

[B34] Moritz ML Ayus JC New aspects in the pathogenesis, prevention, and treatment of hyponatremic encephalopathy in children Pediatr Nephrol 25 7 1225 1238 10.1007/s00467-009-1323-6 PMC2874061 19894066

[B35] Remer T Neubert A Maser-Gluth C Anthropometry-based reference values for 24-h urinary creatinine excretion during growth and their use in endocrine and nutritional research Am J Clin Nutr 2002 75 3 561 569 11864864 10.1093/ajcn/75.3.561

[B36] Steiner RW Interpreting the fractional excretion of sodium Am J Med 1984 77 4 699 702 10.1016/0002-9343(84)90368-1 6486145

[B37] Maesaka JK Imbriano LJ Ali NM Ilamathi E Is it cerebral or renal salt wasting? Kidney Int 2009 76 9 934 938 10.1038/ki.2009.263 19641485

[B38] Burrows FA Shutack JG Crone RK Inappropriate secretion of antidiuretic hormone in a postsurgical pediatric population Crit Care Med 1983 11 7 527 531 10.1097/00003246-198307000-00009 6861500

[B39] Fomon SJ Ziegler EE Renal solute load and potential renal solute load in infancy The Journal of pediatrics 1999 134 1 11 14 10.1016/S0022-3476(99)70365-3 9880442

[B40] Bland JM Altman DG Statistical methods for assessing agreement between two methods of clinical measurement Lancet 1986 1 8476 307 310 2868172

[B41] Mallie JP Bichet DG Halperin ML Effective water clearance and tonicity balance: the excretion of water revisited Clin Invest Med 1997 20 1 16 24 9013040

[B42] Wolf AV Mc DM Apparent and osmotic volumes of distribution of sodium, chloride, sulfate and urea Am J Physiol 1954 176 2 207 212 13124520 10.1152/ajplegacy.1954.176.2.207

[B43] Edelman IS Leibman J Anatomy of body water and electrolytes The American journal of medicine 1959 27 256 277 10.1016/0002-9343(59)90346-8 13819266

[B44] Verbalis JG Goldsmith SR Greenberg A Schrier RW Sterns RH Hyponatremia treatment guidelines 2007: expert panel recommendations Am J Med 2007 120 11 Suppl 1 S1 21 17981159 10.1016/j.amjmed.2007.09.001

[B45] Au AK Ray PE McBryde KD Newman KD Weinstein SL Bell MJ Incidence of postoperative hyponatremia and complications in critically-ill children treated with hypotonic and normotonic solutions The Journal of pediatrics 2008 152 1 33 38 10.1016/j.jpeds.2007.08.040 18154895

[B46] Neville KA Sandeman DJ Rubinstein A Henry GM McGlynn M Walker JL Prevention of hyponatremia during maintenance intravenous fluid administration: a prospective randomized study of fluid type versus fluid rate J Pediatr 156 2 313 319 e311-312 10.1016/j.jpeds.2009.07.059 19818450

[B47] Eulmesekian PG Perez A Minces PG Bohn D Hospital-acquired hyponatremia in postoperative pediatric patients: prospective observational study Pediatr Crit Care Med 2010 11 4 479 483 20124948 10.1097/PCC.0b013e3181ce7154

[B48] Au A Bell MJ Prevention of hospital-acquired hyponatremia in children: Are hypotonic solutions safe? Pediatr Crit Care Med 2010 11 4 528 529 10.1097/PCC.0b013e3181d50366 20606555

